# "For someone who's rich, it's not a problem". Insights from Tanzania on diabetes health-seeking and medical pluralism among Dar es Salaam's urban poor

**DOI:** 10.1186/1744-8603-6-8

**Published:** 2010-05-04

**Authors:** Marie Kolling, Kirsty Winkley, Mette von Deden

**Affiliations:** 1 Department of Anthropology, Faculty of Social Science, University of Copenhagen, Denmark; 2 Diabetes & Mental Health Unit, King's College London & Institute of Psychiatry, UK

## Abstract

The prevalence of chronic non-communicable disease, such as type 2 diabetes mellitus (T2DM), is rising worldwide. In Africa, T2DM is primarily affecting those living in urban areas and increasingly affecting the poor. Diabetes management among urban poor is an area of research that has received little attention. Based on ethnographic fieldwork in Dar es Salam, the causes and conditions for diabetes management in Tanzania have been examined. In this paper, we focus on the structural context of diabetes services in Tanzania; the current status of biomedical and ethnomedical health care; and health-seeking among people with T2DM. We demonstrate that although Tanzania is actively developing its diabetes services, many people with diabetes and low socioeconomic status are unable to engage continuously in treatment. There are many challenges to be addressed to support people accessing diabetes health care services and improve diabetes management.

## Introduction

Diabetes affects approximately 246 million people worldwide[1] and has become a major threat to global public health[2]. In Africa, the prevalence of diabetes has increased significantly and the International Diabetes Federation (IDF) Atlas 2006 reports an overall prevalence of diabetes at 3.1%, affecting a total population of 10.4 million people; a huge number despite a lower prevalence than Europe, 8.4%, and North America, 9.2% [1].

In this paper we seek to explore the global diabetes epidemic from a local perspective by investigating the challenges to diabetes management among urban poor in Dar es Salaam, Tanzania. Since the 1980s, Tanzanians have witnessed a rapid rise in chronic disease such as T2DM. Incidence of T2DM has gone from among the lowest in the world to an estimated 909,600 out of Tanzania's approximately 41 million people and prevalence is expected to increase by 50% within the next 20 years[3,4].

Diabetes is known to be more common in some African countries rather than others, notably in Northern and Southern African nations, and within countries levels are higher in urban areas compared with rural areas, which is also the case of Tanzania[5]. The prevalence in urban versus rural Tanzania is 5.8% and 1.7%, respectively[3]. Diabetes in Africa is often perceived as predominantly affecting the affluent or those moving up the socioeconomic ladder and until relatively recently, diabetes in Africa was considered rare[3]. However, incidence is increasing in low and middle-income nations and increasing among the poor[1,6], matching what has long been known; that low socioeconomic status equals poor health[7].

Given its chronic nature, most diabetes care takes place in the everyday life of the person with diabetes, their private sphere, rather than in the public sphere of the health care system. Studies from the patient's perspective with an emphasis on self care practices are therefore important in order to understand factors affecting diabetes management in Africa. Using an ethnographic approach to conduct fieldwork in Dar es Salaam, Tanzania, we proposed to try and unfold the complexity of the causes and conditions for poor diabetes management and investigated how people cope with the illness in a resource deprived environment where access to and availability of the means to control diabetes are limited. In this paper we set out to describe and analyse (1) the structural context of public diabetes services in Tanzania; (2) the current status of biomedical health care and ethnomedical health care; and (3) the experiences that diabetes patients have and the actions they take accordingly.

## Methodology

Two months of ethnographic fieldwork was carried out in 2008 among urban poor with T2DM in Dar es Salaam, Tanzania. The fieldwork was conducted by MK and MvD. In 2009 a brief follow-up visit was conducted by MvD. The ethnographic study will be referred to in this paper as the 'Tanzania study'.

### The setting and the informants

Dar es Salaam is the largest city in Tanzania with a total population of 2.5 million as of the official 2002 census. Dar es Salaam has experienced rapid urbanisation over the past decade, overwhelming the city's infrastructure and services[8,9]. The city has an estimated unemployment rate of nearly 30 percent with many employed in the informal sector[8].

The informants in the Tanzania study lived in impoverished areas of Dar es Salaam, some at the outskirts of the city, and all belonged to the lower socioeconomic class. The 29 primary informants were aged approximately between 32 - 70. Some had recently been diagnosed with T2DM and some had been diagnosed up to 20 years ago and their experiences with the illness were therefore diverse. Many informants had stopped working due to their illness. Four of the informants were formally employed at the time of inquiry and some were engaged in irregular income generating activities such as occasional informal business from their homes or as street vendors. The majority of informants had completed primary school and four informants had a higher level of education which matches the figures from the 2004-2005 Tanzania Demographic and Health Survey that states only 10 percent of the population has more than primary education[10]. The informants had different religious and ethnic backgrounds and some were migrants. Our findings are hereby specific to a particular social group - urban poor with T2DM living in Dar es Salaam - and may not be generalisable to other populations even within Tanzania. To contextualize the life situations of the primary informants, 11 secondary informants were also interviewed such as family members, health care professionals, traditional healers, an informant's employer, policymakers, and employees of the Tanzania Diabetes Association.

### Data collection and analysis

The methodological entry point for the fieldwork was three public diabetes clinics located in each of the three districts in Dar es Salaam. The fieldwork was initiated at public diabetes clinics to ensure that primary informants were diagnosed with T2DM and were from lower socioeconomic classes. At the diabetes clinics, contact was established with informants by spending time in the waiting area and engaging in conversations while they were waiting for their appointments. Following the conversations, some patients were invited to participate in a focus group and with other patients, home visits were arranged. Two focus groups were conducted at the Tanzania Diabetes Association before initiating the home visits in order to obtain a broad insight into how diabetes was perceived and managed. With those participants from the focus groups who wished to further participate in individual interviews, home visits were also arranged. Through informants' social networks contact was gained with other informants who were not receiving biomedical diabetes treatment.

The main fieldwork sites were the communities of the informants since the research objective was to investigate how people cope with diabetes in their everyday lives and to do so from the perspective of the patient. The research was hereby centred around what Kleinman (1980)[11] has categorised as the *popular sector*. The *popular sector *refers to the treatment that takes place outside of the sphere of the biomedical health care system. This is where the illness is first encountered, symptoms evaluated and decisions on what to do about it are initiated. Kleinman has termed the sphere of the biomedical health care system the *professional sector *and the sphere of ethno-medicine the *folk sector*. Given that diabetes is a chronic illness, people spend much more time taking care of their illness in the *popular sector *than in the *professional sector *or in the *folk sector *and the *popular sector *can therefore be conceived as the most significant arena of care[11,12].

Overall, a variety of methods were employed during the fieldwork which included semi-structured and in-depth interviews, focus groups, observation at three public diabetes clinics and participant observation in the local context of the daily lives of the primary informants in Dar es Salaam. Follow-up interviews were conducted and home visits were repeated with key informants. The research design adhered to general ethical guidelines[13]. Verbal consent was gained from all the informants in the study prior to the interviews. The informants were assured that their participation was voluntary and would not interfere with their diabetes treatment at the clinic, that the data would be handled in a confidential manner and that their names would not be used in any publication or presentation. Hence the names that appear in this paper are pseudonyms.

The informants' native language was Swahili and the two focus groups and the majority of individual interviews were therefore conducted with interpreters from the University of Dar es Salaam. The interpreters were carefully instructed about ethnographic interview techniques and took on the task of introducing MvD and MK to local culture and customs. Some of the information provided by informants might be lost in the situational translation and all the interviews were therefore recorded and the majority transcribed in English by the interpreters who had undertaken the interview. MvD and MK transcribed those interviews conducted in English without interpreters. Upon transcription, basic demographic information about the informants was clarified.

The analytical process was dynamic and multifaceted, drawing on the interview recordings, transcriptions, field notes, scratch notes, the coding of themes, mind maps and shared fieldwork experiences that were discussed at length in relation to theoretical insights. Theories for the analysis were primarily drawn from the fields of medical and political anthropology. De-Graft Aikin's concept of *illness action *was central to the analytical approach since it places the individual with an illness within a wider social, material and political context[14]. The main goals of the analysis were to outline the life stories and illness experiences of the informants and identify what was at stake for them.

## Findings

The presentation of the findings from the Tanzania study is organised into the three themes: (1) The structural context of biomedical and ethnomedical care; (2) health-seeking; and (3) ethnomedical health-seeking.

### The structural context of biomedical and ethnomedical health care for people with diabetes in Tanzania

Recently, a network of diabetes clinics had been established throughout Tanzania which had provided approximately 100,000 people access to affordable diabetes treatment and health education. These diabetes clinics had been established by the Tanzania Diabetes Association in collaboration with the Tanzanian Ministry of Health (MoH) and other partners and were run by district, regional, and referral hospitals. Consultations were free of charge and clinical assessment procedures such as weight, blood pressure, and blood glucose level were heavily subsidised and cost 1000 Tsh. (1 USD at the time). The poorest patients paid nothing because of the national exemption waiver system, although in practice an evaluation showed that many of those who should be eligible for exemptions did not have access to it[15], which was also our experience. In the past, before these diabetes clinics were established, diabetes services were provided at regional hospitals by staff with no specialist diabetes training. Specialist diabetes care was only available at five referral hospitals, run by a small number of consultant diabetes physicians and diabetes educators and hence access to diagnosis and treatment particularly for people in Tanzania's rural areas was extremely limited[16]. At the time of the fieldwork, the Tanzanian Ministry of Health was formulating a national strategy on non-communicable and chronic diseases that once completed and implemented should further improve health interventions in this area (Interview MoH, Malene Krag Petersen, March 2008).

The biomedical treatment offered in Tanzania has improved, but there is still much to be done. The three public clinics at the district hospitals in Dar es Salaam that MK and MvD visited were full of patients waiting for their consultation, and across the diabetes clinics the increase in patients and a shortage of qualified personnel affected provision of timely and appropriate treatment[16]. A nurse at the diabetes ambulatory at the National Hospital told us that the increase in patients meant that the frequency of appointments was reduced, lowering the quality of the treatment offered to patients (Interview February 2008). Furthermore, at the district hospitals insulin and other medicines had previously been available heavily subsidised, but at the time of inquiry Tanzania had had a nationwide shortage of insulin for more than two years (Interview MoH, Krag Petersen, March 2008). In consequence, many people had to buy the medicine at private pharmacies where it was still available, but at a high cost. This was the case for a female informant, Helen, 52. We spent a day accompanying Helen in need of buying insulin which was part of conducting participant observation in the local context of informants' daily lives.

#### Case 1. Helen's illness experiences

Helen left her home on the outskirts of Dar es Salaam in the morning and commuted on crowded *dala-dala *minibuses through the bumpy dirt roads to the city centre. During the bus rides she used her diabetes to gain a seat on the *dala-dala *or a better place in a queue. MvD and MK met Helen the first day of the fieldwork at one of the public diabetes clinics. We had arrived an hour before the clinic opened and the waiting area was already full of patients and the relatives accompanying them. We had been talking to a few patients with the assistance of our interpreter when Helen approached us and wanted to talk to us. One of the things she wished to express was that diabetes patients were overlooked compared to patients with HIV/AIDS who received free treatment and medication which she felt diabetics should receive as well. She pointed out the HIV/AIDS clinic which had recently been renovated and which was located across from the diabetes clinic. It was an impressive white building in comparison to the diabetes clinic which was in fact a container transformed into a clinic and located at the margins of the district hospital. Helen, we learned, was one of the patients at the district clinics who had managed to make use of the exemption waiver system mentioned above and did not pay for the medical check-ups. These illness actions; using her diabetes to gain a seat on the bus, a better place in the queue or managing to make use of the exemptions, made Helen appear resourceful. Yet, the day we accompanied her looking for insulin, she spent the whole day going from pharmacy to pharmacy to find insulin at an affordable price, at which she failed, and returned home empty handed. Going without insulin from time to time was nothing unusual to Helen which proved her difficult situation in spite of our impression of Helen as one of the more resourceful patients at the diabetes clinics.

It was our experience that many of the informants in the Tanzania study, like Helen, did not take their medicine regularly because they were unable to purchase the medicine. It was not only insulin that was unavailable at the hospital pharmacies. Also oral tablets were in shortage as a female informant explained (Rose, 54):

"Most of the time, the hospital pharmacy never has the tablets. Then I have to go to the private pharmacy and I mostly buy there since the store at the hospital doesn't have the medicine."

The shortage of subsidised insulin and oral medication was evident from the informants' accounts. Providing people access to insulin and oral medication as well as diabetes services is important in order for them to engage continuously in treatment and a female informant addressed the problem of gaining access to medication and treatment in a focus group discussion in this manner. She said (Zalika, 63):

"This problem is something that happens to poor people. For someone who's rich, it's not a problem."

In Tanzania the medical sector is pluralistic with biomedical health care systems and ethnomedical health clinics existing side by side and offering different *explanatory models*, a term coined by Kleinman[11], concerning the causes of diabetes and means of effective treatment. Ethnomedical health clinics of various ethnomedical approaches are dispersed all over the city of Dar es Salaam and many are placed next to or close by a biomedical clinic or hospital. MK and MvD interviewed a herbalist healer whose clinic was located further down the road from one of the district hospitals. He explained that he usually sent his patients to a diabetes clinic for diagnosis if he suspected the patient suffered from diabetes. Once the person had been diagnosed, the healer would initiate a herbal treatment which he claimed was able to cure any patient within one month. In our experience, for many people with diabetes such ethnomedical cure provided an attractive alternative to the prospect of lifelong biomedical treatment as will also be explored below.

### Health-seeking in Dar es Salaam

The majority of the informants in the Tanzania study was not financially independent and people's self-care practices have to be seen as a collective praxis and not simply as an individual matter. The family members in the household, particularly the members of the nuclear family, provided care and treatment to the person afflicted by diabetes in terms of acquiring medicine, accompanying the person to health care services, knowledge sharing, and upholding a healthy diet since buying food was a collective matter especially in times of financial hardship. This mutual involvement and care performed by the family network supports existing anthropological knowledge on the function of the kinship system in which the kinship groupings are the most important social units for most people across the African region. The kinship system is characterised by social dependency and mutual obligations between kin and has a high degree of self-reliance in coping with disease and illnesses, as patterns of family treatment and care are deeply embedded within this wider kinship system[17]. Although the family network was a fundamental support and an enabling factor for the actions that people took in relation to their illness, the interdependent character of the relations among relatives also had constraining consequences for these actions. Most of the informants' self-care practices were severely constrained because the needs of other relatives also had to be met. Many of the informants had to adjust their illness actions to the needs of other relatives which Haiba's illness experience illustrates.

#### Case 2. Haiba's illness experiences

Haiba, a woman aged 54, lived in a semi rural village on the very outskirts of Dar es Salaam in a household with her 12 family members. Five of these were her children. Her grown-up daughter was seriously ill from tuberculosis and required demanding daily care and medicine. Haiba was not alone in her care-giving role. Other females in the households assisted her, but being the mother of the ill daughter, Haiba had the primary care-giving responsibility for her daughter[12] and since more than one person within the household was sick, it entailed choosing whom to help. Haiba chose her daughter's health to her own. This meant that she occasionally went without insulin in order to ensure that there was enough money within the household to buy her daughter's medicine.

Haiba's story illustrates how the needs of other relatives also had to be met which often compromised the needs of the person with diabetes. It further illustrates how the double burden of disease puts tremendous pressure on the remaining relatives[17] and how prioritising was a continuous process that severely jeopardised the sustainability of the long-term treatment schemes necessary for survival.

In terms of financial support, our findings also show that there were limits to the obligations of kin. People were seeking out other ways of getting support as the mobilisation of resources had been extended to wider social networks. Many of the informants drew on support within their social network by approaching friends, colleagues or people within their local communities. A male informant explained to us in the following part of a home interview (Haamed, 35):

"*Sometimes, when things get hard, I go to the business where I used to work and the colleagues they give me something. It is not fair every time to go to my brother*."

Similar observations have been revealed in a recent study from western Kenya[18]. This tells us of the need to revise the role of the family in general and include alternative support possibilities in our understanding of the conditions for illness actions in particular.

Furthermore, we argue that the diminished capacity due to hardship and deaths within the networks made the dynamics of illness action discontinuous as the intentions of the primary agent may be disrupted by other events. Such events may be the sudden death of the household provider or other relevant family members. The unpredictability and discontinuity of the life condition of the people with diabetes in the Tanzania study are illustrated through the story of one of the informants, Fatima, a woman of 32.

#### Case 3. Fatima's illness experiences

MK and MvD met Fatima during the first days of observation at one of the diabetes clinics. While most patients at the clinic were accompanied by a relative, Fatima was alone and sat to herself. She appeared introverted quite opposite to Helen who approached us at the clinic, wanting to talk. Fatima could not afford the medical check-up and only attended the doctor to obtain her prescriptions. Fatima lived with her younger sister in a small rented shack in an impoverished neighbourhood in Dar es Salaam. Fatima had moved to the city after her husband had committed suicide, her sister who once had employed Fatima in her restaurant had died from AIDS and her father had died from illness.

Our first home visit was characterised by a tense atmosphere as the hardship of life was obvious and constantly emphasised throughout the interview. When we contacted Fatima to arrange a follow-up interview, she had left Dar to visit the village where her mother lived with two of Fatima's children. We assumed that such a journey was an unaffordable expenditure for her since neither Fatima nor her sister had a job and they were both financially completely dependent on their older brother who lived in Dar. Unfortunately, we learned that Fatima's visit to her village was due to dire circumstances. A brother had passed away and she went to take part in the funeral. When we asked her how she managed to pay the bus fare, she explained that her relatives had rented a car to transport the body of the deceased. Realising that the brother was not living in the village but in Dar, we asked if the deceased brother was the one supporting her. Tragically, it was. While at work, robbers had attacked and shot him. With this information the interview took a whole different turn than planned since it was obvious that this sudden death in the family was bound to have a profound impact on Fatima's life condition. The brother had also helped support her children, and in fact the majority of Fatima's family members used to depend on the deceased brother. Apart from paying her food and housing, Fatima's brother had also been paying for one of the three medications she had been prescribed at the clinic and with his sudden death there did not seem to be any chances of her receiving more medical treatment.

We interviewed Fatima during a time of mourning which traditionally lasts 40 days after the funeral. When the mourning was over, her relatives were to decide what to do; not only about Fatima's situation, but also about the brother's wife and children and others. The outcome of their decisions was uncertain.

Fatima's situation was one of the most vulnerable among the informants and her story sums up the unpredictability and discontinuity of the life condition of the people in the Tanzania study. Fatima's vulnerable internal health conditions combined with the extreme vulnerability of external conditions disrupted all illness actions and left Fatima with few choices which may have had severe consequences for her future.

The lives of the informants were characterised by poverty, insecurity, uncertainty, and hence unpredictability. According to our observations, this suggests that the possibilities for health-seeking behaviour were often assessed on a day-to-day basis. This made illness action vulnerable to disruption and tactical in character. This furthermore opposed the long-term strategies promoted by health professionals for the treatment of diabetes and often favoured the short-term treatment found among herbalist healers, claiming to cure their illness. This will be explored below.

### Ethnomedical health-seeking

It was also our experience that medical pluralism increased the range of therapeutic choice and complicated health-seeking behaviour which Hardon et al. also have argued[19]. From interviews with health care professionals at the public diabetes clinics, MK and MvD were told that most or many of their patients had tried, or took, some sort of traditional therapy. Many of the informants in the Tanzania study had some contact, directly or indirectly, with herbalist treatment concomitant with attending treatment at biomedical diabetes clinics. Several also confirmed that they had interrupted their biomedical treatment in order to follow an ethnomedical treatment. From interviews, we found that the primary reason for using ethnomedicine was that it was less expensive and thus more affordable compared with the price of medicine at private (biomedical) pharmacies. Many informants had not attended a herbal healer, but their relatives had bought the herbal medicines for them. This was the case of Rose, 54:

"*The first time I tried to use some traditional medicine it was my sister who brought it to me. Then, when I went to church there was a relative of our pastor from the church who knew of a medicine that cures diabetes, but again it didn't help me anything either. Now, I'm using another kind of medicine which I don't know where it comes from, but it is packed in a yellow plastic bottle and it's written that it contains aloe vera. When I take this last medicine, I don't feel so tired as before and so it reduces the tiresome that I normally have. It's my daughter who buys this one for me at her work*."

Another reason for using ethnomedicine was that herbalist healers, or others selling ethnomedicine, claimed it would cure diabetes. This was also the motivation for Rose to continue experimenting with herbal medicines which she also expressed when we asked her what advice she had been given concerning the herbal medicine she was taking. She said:

"I was told that when I take this, it will help curing many diseases which are in the body. You have to take six plastic bottles of this size in order to be completely okay. It really helps!"

In some cases beliefs in herbal medicine had a profound impact on the treatment of the person with diabetes, but we also found that informants merely saw the herbal medicine as an opportunity which they had to try out. Rose's experience illustrates this as she kept experimenting with new ethnomedicines, hoping to be cured, while still taking the oral tablets prescribed by the doctor at the diabetes clinic at one of the district hospitals. However, these tablets were not taken uninterrupted:

"I usually take them as required by the dose and when they finish I beg for assistance of money from relatives and friends. But sometimes it might happen that I stay for two three days without the medicine until I collect enough money to buy the medicine."

Another informant, Hasani, 35, had also been advised by a relative to try herbal medicine. He had hoped it would improve his condition as he was dissatisfied with the oral tablets he had been prescribed by a doctor at a private diabetes clinic he attended. Hasani had migrated to Dar es Salaam when he was 16 and had lived for many years with an uncle who had recently passed away due to diabetes. It was this uncle who had advised Hasani to try herbal medicine. This passage about Hasani's experience with ethno-medicine is from our second interview with him:

"My uncle, who was the one who received me when I came to Dar and employed me in his grocery, he was also the one I told you last time that he advised me to use the aloe vera herbs when he discovered that I had the problem. He had the aloe vera plants in his farm and so he advised me to use it. I had started taking tablets, but the tablets gave me headaches. I was given tablets to use for one month and my uncle advised me that I finished using the one month tablets and switched and tried to use herbs like aloe vera and some other herbs whose names I don't remember. With the tablets my weight had continued to fall until the point I reached 54 kgs. So when I started using the herbs, my weight begun to re-gain back until it reached 70 kgs. I then stopped taking the medicine and stayed for almost three months without taking any medicine at all. Then I started feeling ill again with the same symptoms that I felt at the beginning of the problem. When I decided to re-use the Aloe Vera the doctor told me that this aloe vera may be poisonous and could result in kidney failure. That was when I decided to go to the district hospital to open my first clinic card."

Ethnomedical treatment often relieves people short-term; physically by easing the symptoms and psychologically by imposing hope of a cure, which Hasani's experiences also show. Unlike Rose, Hasani stopped taking the herbal medicine once he was told it could be poisonous. The tactical character of illness action made ethnomedical treatment seem even more favourable to biomedical treatment and when placed in a position of financial hardship, the long-term future was blurred to most of the informants and unpredictable which led them to make decisions concerning their health based on immediate opportunities and obstacles.

## Discussion

The discussion is organised into the three themes: (1) biomedical care; (2) ethnomedical care; and (3) health-seeking. The findings are discussed in relation to other African contexts.

### Biomedical care

With the rise in diabetes prevalence in the African region, the Tanzania study provides insights into the challenges posed by the biomedical health care system; a system struggling to treat a growing number of patients with diabetes. Most people with diabetes that were interviewed seemed to value biomedical treatment but experienced difficulties engaging continuously in treatment due to geographical and financial constraints as well as poor physical health. In Tanzania, specialised diabetes clinics are concentrated in urban areas and living far from them is costly. The price of bus fares frequently prevents patients from attending treatment and may undermine continuous engagement in care. Even for those living in the urban areas, geographical and financial constraints may contribute to discontinuity in their diabetes care, but also the patient's constant and changing physical health can have a direct negative influence on their treatment attendance. The Tanzanian data revealed that blurred vision, muscle pain, and impaired memory, all common symptoms of diabetes, could prevent a patient from commuting to the diabetes clinic for treatment. Diabetes does not always cause symptoms, but when present they often indicate declining health[11]. This means that in times of worsened physical health, the person may not have enough strength to seek treatment and therefore stays at home and thus lacks adequate care.

Although biomedical treatment for diabetes in Tanzania is over-subscribed and often inadequate, it is more organised than in many other African countries and the MoH and other private or non-governmental agents are actively striving to develop and implement services and train specialist staff. In comparison, in Ghana, there are only two specialist diabetes centres. Both are in the urban south, in the capital and in the second largest city, which means that people living with diabetes in the north and rural south do not have access - or face great difficultly accessing - specialist diabetes care. A lack of access to biomedical services may delay treatment, and mis-(self)-diagnosis may predispose late presentation and worsen diabetes outcomes. Many informants in the Tanzania study did not receive the T2DM diagnosis until the illness had progressed and complications had started to manifest, leading to a dramatic event that required hospitalisation where the cause of their ill health was finally identified.

There is still much to be done with shortages of services and low-cost medicines, meaning that people with diabetes have to consider all their available resources for treatment.

### Ethnomedical care

Anthropological research suggests medical pluralism is pervasive across the African region [20-22]. Medical pluralism is a key feature in chronic disease experiences[23] and there are specific accounts of people with diabetes accessing pluralistic medical care in addition to the Tanzania study such as in Ghana and Cameroon [23-25].

Ethnomedicine is sophisticated and highly organised in many African countries. Healers offer a cure, something biomedicine cannot, and low cost treatment. People with diabetes may use ethnomedicine parallel with biomedicine, which the Tanzania data also demonstrated. Likewise, the work of de Graft Aikins[23] shows that pluralistic health care is the norm in Ghana and is completely embedded within the culture, making the two systems interdependent. People with diabetes may use ethnomedicine concurrently with biomedicine but offered a choice, many would prefer biomedical drugs and diet treatment of their diabetes, which seems to fit their (more or less) biomedical view of what diabetes is. However, because of poor resource availability, ethnomedicine might be tried at least once for hope of cure or perhaps to assuage other family members. More choices within health-seeking expand the range of possibilities for treatment but, as could also be observed in the Tanzania study, may lead to further complications and possible deaths. Researchers attribute complications and high mortality to poor medical management and harmful self-care practices, including use of ethnomedicine[26].

### Health-seeking

Analysis of the Tanzanian data among urban poor in Dar es Salaam showed that the illness had a major impact not only on the person with diabetes, but also on the family who had the primary responsibility of caring for ill relatives[17]. Poverty was the fundamental life condition for the informants in this study and the socioeconomic impact of diabetes management made the mobilisation of the family's resources a necessity and diabetes self-care practices a collective practice and not just an individual matter. The family is the major principle around which African kinship groupings are organised and the most important social unit for people in Tanzania and across Africa[17]. Consequently, living with diabetes or caring for someone with diabetes is very much a family matter in an African context. The family was therefore providing the primary support and care for the informants in this study.

Despite the positive and enabling character found within the family system in Tanzania, the interdependence also had constraining consequences for people's illness actions. We reported how the death of a family member who supports the diabetic relatives as well as others within the family had direct fatal consequences for the informant's access to medicine and adequate diabetes diet. This was Fatima's brother to whom Fatima had turned after the loss of her husband and father and on whom she was completely dependent. She was unable to support herself due to diabetes. This shows how the scarcity of material resources within the family is crucial to the kind of action taken and demonstrates how vulnerable and insecure people's access to diabetes care in Tanzania is. This forces the person with diabetes to act tactically and this is illustrated by their active use of affordable herbal (ethno)medicine. We also report how a mother would deny herself insulin in order to provide her daughter with TB medication, highlighting the problem of continual prioritisation brought about by poverty.

Much anthropological analysis has tended to privilege the positive and harmonious aspects of kinship, presenting it as intrinsically desirable. However, the social dependency and mutual obligations among kin also embody negative aspects[27] and may influence diabetes management. It is the diminished capacity of the family to provide care and treatment for diabetic family members, together with the impact of HIV/AIDS, which have worn families thin. This suggests a need to expand existing anthropological theory on the function of the kinship system as new limits are drawn to the obligations of kin and people are currently seeking other ways and beyond the family networks to receive support.

Finally, we argue that the diminished capacity due to hardships and deaths within the networks makes the dynamics of illness action discontinuous as the intentions of the primary agent may be disrupted by other events. Consequently, living with diabetes under such circumstances makes diabetes management extremely difficult.

## Conclusions

From the Tanzania study several of the potential conditions leading to poor diabetes management were identified at a subjective, inter-subjective and structural level. Despite the limited scope and sample of the Tanzania study, our findings provide an insight into how living with diabetes in a resource constrained environment has major implications for diabetes health-seeking, often leaving people with few choices that may have severe consequences for their future health.

In Africa, the rising burden of chronic non-communicable disease such as diabetes, alongside the continued burden of communicable diseases, poses new challenges. Now this doubled threat of disease increases the pressure on relatives as well as the burden on health services and this is the current development in Tanzania. With diabetes prevalence in Tanzania on the rise and increasingly affecting people of low income, particularly in urban areas, Tanzania appears to be ahead of many other African countries in its development of diabetes services. However, it is clear that more resources and innovative health interventions are needed if Tanzania is to tackle the rising chronic non-communicable disease burden. This is even more salient for countries which have yet to develop a national policy on this issue.

## Authors' information

MK is a post-graduate student of anthropology at the University of Copenhagen, Denmark. In her thesis she investigates the illness experiences of men with T2DM and gendered responses to diabetes for which she conducted five months' ethnographic fieldwork in Northeast Brazil in 2009.

KW is lecturer in diabetes and psychology at King's College London & Institute of Psychiatry. She is also a diabetes specialist nurse and health psychologist. Her primary research interest is to develop biopsychosocial interventions to improve adverse outcomes in people with diabetes.

MvD is a post-graduate student of anthropology at the University of Copenhagen, Denmark. Currently, she has undertaken a five-month study on individual and family diabetes management among African migrants in South East London and also takes an interest is people's management of communicable illnesses.

## Competing interests

MK and MvD were sponsored by Novo Nordisk for their Tanzanian fieldwork.

KW has received lecturing fees from Eli Lilly.

## Authors' contributions

All authors conceived of the topic and structure of the paper. MK and MvD contributed data on Tanzania and drafted the manuscript. KW contributed the review on diabetes in Africa and the draft synthesis. All authors contributed to data synthesis and read and approved the final manuscript.
